# False-Positive Radioiodine Uptake in Simple Ovarian Cyst in a DTC Patient: A Case Report

**DOI:** 10.3389/fonc.2021.665135

**Published:** 2021-05-31

**Authors:** Tao Wu, Xuefeng Zhao, Huiqin Xu

**Affiliations:** Department of Nuclear Medicine, The First Affiliated Hospital of Anhui Medical University, Hefei, China

**Keywords:** false-positive, radioiodine, simple ovarian cyst, thyroid cancer, single photon emission computed tomography/computed tomography

## Abstract

Abnormal radioildine uptake can be caused by various pelvic lesions in differentiated thyroid cancer patient. Here we presented an abnormal uptake in the left side of the pelvic cavity on postablative I-131 scintigraphy in a 51-year-old woman with history of stage T1aN1M0 papillary thyroid cancer. The SPECT/CT fused imaging revealed the lesion in the left ovary. Laparoscopic bilateral adnexectomy showed a left ovarian mass (5 cm) and pathologic finding showed a simple ovarian cyst. The nonstimulated Tg immediately decreased to 143 ng/ml after bilateral adnexectomy 3 days later and to 0.109 ng/ml after 4-month follow-up. Timely intervention measures are very necessary for patients with ovarian cyst with abnormally elevated Tg level.

## Introduction

Radioiodine (I-131) diagnostic whole body scan (WBS) is a very effective test in the treatment strategy of differentiated thyroid cancer (DTC). Single photon emission computed tomography (SPECT) is the most commonly used device to identify the residual thyroid and distant metastases. This is mainly due to the fact that most DTC cells can accumulate iodine through the action of the sodium-iodine symporter (NIS) (1). Despite its high sensitivity, its specificity is poor due to the lack of detailed anatomical localization in planar imaging (2). Because false-positive images often occur due to physiological ingestion, such as retention of radioiodine in the gastrointestinal tract, salivary gland uptake, and lesions in other parts of organs, such as mucinous cystadenoma (3), struma ovarii (4), endometrial cyst (5), gallbladder stones (6), hepatic cyst (7), renal cyst (8), nabothian cyst (9). SPECT combined with X-ray computed tomography (SPECT/CT) fusion imaging can provide additional information to distinguish between metastatic lesion and other abnormal uptake. Here we presented a clinical case with local pelvic abnormal uptake, where SPECT/CT accurately located the cystic lesion of left ovary which was pathologically confirmed to be a simple ovarian cyst.

## Case Presentation

**Figure 1:**
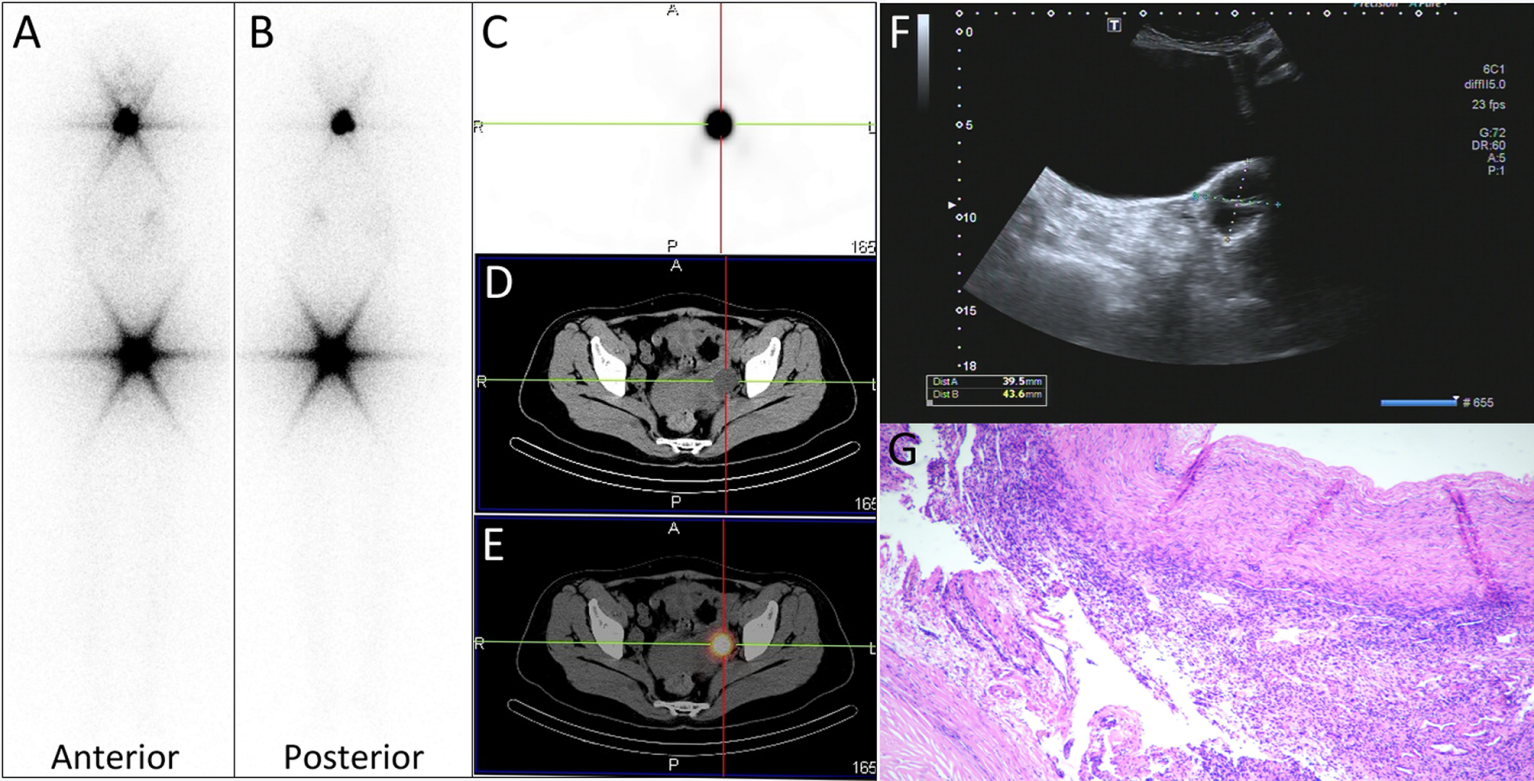
**(A, B)** panels images showed intense radioiodine uptake in thyroid bed in the neck and unexpected uptake in the left side of the pelvic cavity. **(C–E)** Axial tomography images demonstrated the unexpected uptake corresponded to a ovarian cyst of approximately 3.2 × 2.4 cm in size. **(F)** The pelvic ultrasonography showed a 4.3 × 4.0 cm liquid area with internal separation in the left ovary. **(G)** Pathologic finding (hematoxylin and eosin staining ×40) showed a simple ovarian cyst.

On March 5, 2018, a 51-year-old woman went to our hospital with the chief complaint of “right thyroid nodule” for subtotal thyroidectomy and central lymph node dissection. The preoperative serum thyroglobulin (Tg) level was 118.28 ng/ml (3.5–77 ng/ml). Postoperative pathology confirmed bilateral multifocal papillary thyroid carcinoma with a stage T1aN1M0 (four lesions about 0.2–0.6 cm in diameter, and one metastatic lymph node in the right central region, and no other metastatic lesions). Five months after surgery she underwent a radioiodine therapy as remnant ablation and adjuvant (10) with an activity of 3.7 GBq (100 mCi) of I-131. The nonstimulated Tg level was 9,572 ng/ml 1 month before radioiodine remnant ablation. After 18 days of thyroid hormone withdrawal, her serum Tg level was 11,488 ng/ml with antithyroglobulin antibodies (A-Tg) of 37.9 IU/ml (manufacturer cutoff level <115 IU/ml, limit of quantitation not available), and the thyroid-stimulating hormone (TSH) concentration was 7.91 mIU/L (0.27–4.20 mIU/L). The post-treatment I-131-WBS (**Figure 1A**, anterior view; **Figure 1B**, posterior view) performed 3 days after I-131 administration revealed intense radioiodine uptake in thyroid bed in the neck and unexpected uptake in the left side of the pelvic cavity. Further SPECT/CT fused imaging was performed on the symbia T16 scanner (Siemens Medical Solutions) using a 128 × 128 matrix, 1 zoom, 30 s per frame and a CT scan layer thickness of 5 mm to distinguish the source of the pelvic unexpected uptake. The axial images (**Figure 1C**, SPECT; **Figure 1D**, CT; and **Figure 1E**, fusion) demonstrated the unexpected uptake corresponded to a cystic low-density lesion of approximately 3.2 × 2.4 cm in the left adnexa region. The pelvic ultrasonography was then performed, which showed a 4.3×4.0 cm liquid area with internal separation in the left adnexa region (**Figure 1F**). Two weeks later, laparoscopic bilateral adnexectomy was performed under general anesthesia and a 5-cm mass of the left adnexectomy was found intraoperatively. Finally pathologic finding showed a simple ovarian cyst (**Figure 1G**). Three days after surgery, the nonstimulated Tg level immediately decreased to 143 ng/ml from stimulated state (11488 ng/ml).

**Figure 2:**
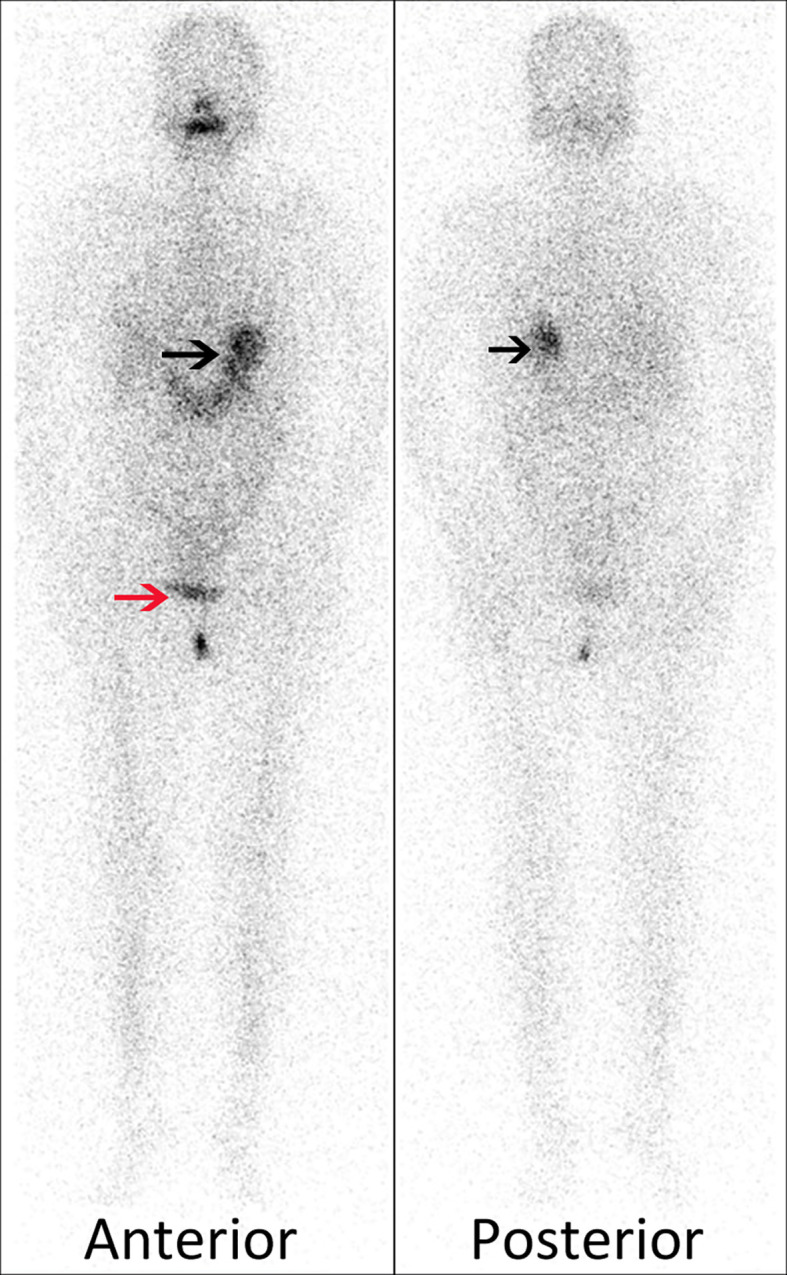
Excepting for the physiological retention of stomach (black arrows) and bladder (red arrow), no radioactive uptake was observed in the neck and pelvic cavity on the diagnostic I-131-WBS.

After a 4-month follow-up, the nonstimulated serum Tg level decreased from 143 to 0.109 ng/ml. After 1 day of administration of 185 MBq (5 mCi) of I-131, excepting for the physiological retention of stomach (black arrows) and bladder (red arrow), no radioactive uptake was observed in the neck and pelvic cavity on the diagnostic I-131-WBS (**Figure 2**). So further SPECT/CT fused imaging was not performed. Up to now, the patient was regularly reexamined for thyroid hormone and Tg level, as well as cervical ultrasound. No abnormal tissue and significant lymph node were observed on the cervical ultrasound images. The Tg level was stable between 0.1 and 0.5 ng/ml after twice L-T4 withdrawal.

## Discussion

Iodine is an essential constituent for the synthesis of thyroid hormones. The uptake of iodine by thyroid follicular cells mainly depends on the expression of NIS in the basolateral membrane. The radioactive isotopes (I-131) of iodine can be quickly absorbed, oxidized and organized by thyroid follicular cells with an effective half-life of 7.3 days due to its similar physical and chemical properties. Based on this principle, radioiodine has been widely used in the diagnosis and treatment of differentiated thyroid cancer.

NIS also slightly expressed in the epithelial cells of other normal tissues, such as salivary glands, lacrimal glands, choroid, gastric mucosa and lactation mammary gland (11). False-positive lesions often appear on I-131-WBS images. In addition, the causes of false positives mainly include physiological retention, contamination of physiological secretions and some non-thyroid related benign and malignant lesions. For instance, biliary duct dilatation can also lead to local hepatic uptake and the metabolism of radioiodinated thyroid hormones can cause diffused hepatic uptake.

SPECT is the most commonly used and sensitive diagnostic tool in clinical practice, but its specificity is poor due to the lack of detailed anatomical localization. But SPECT/CT fusion scanning technology can provide more accurate anatomical location (12), which has significantly improved the accuracy of the differential diagnosis of false-positive lesions. A false-positive finding due to a functional ovarian cyst was first described in 2013 (13). But the Tg level of that patient before radioiodine administration was only 43.48 ng/ml, and the ovarian cyst disappeared after 6-month follow-up without any intervention. However, the stimulated Tg level of this patient in our case was 11,488 ng/ml, and nonstimulated Tg immediately decreased to 143 ng/ml after bilateral adnexectomy under laparoscopy three days later and to 0.109 ng/ml after 4-month follow-up. Although no mutated epithelial tissue was observed, metastatic cystic lesions cannot be excluded because of such high Tg level. This may be the reason for the rapid decrease of Tg after resection of the simple cyst, but which is needed to confirm by further immunohistochemistry. Therefore, timely intervention measures are very necessary for patients with ovarian cyst with abnormally elevated Tg level. To our knowledge, simple ovarian cyst with such high Tg level has not been previously reported in the literature.

As described in the literature, pelvic false-positive lesions were also reported in struma ovarii (4), serous cystadenoma (14), mucinous cystadenoma (15), granulose cell tumor (16), mature teratoma (17), and cystadenofibroma (18). There are no clear mechanisms for these abnormal uptake except for struma ovarii. Besides, other elsewhere benign cyst can also cause radioiodine uptake, such as hepatic cyst (7), renal cyst (8), nabothian cyst (9), spermatocele (19), thymic cyst (20), dermoid cyst (21), and so on. The possible mechanism for the abnormal uptake in renal cyst is linked to an active secretory process by the renal tubule (22), and in functional cyst is passive diffusion of radioiodine into the cyst (23). However, the exact uptake mechanism still needs further exploration and research.

Although differentiated thyroid cancer is a relatively low-grade malignant tumor, distant metastases like lung, liver, and bone are also frequently observed in I-131-WBS images. Therefore, how to differentiate and diagnose these false-positive lesions and prevent these patients from receiving additional internal radiation dose is the focus of clinical practice.

## Conclusion

A false-positive case with such high stimulated Tg level results from a simple ovarian cyst has been presented. The nonstimulated Tg immediately decreased to 143 ng/ml after bilateral adnexectomy three days later and to 0.109 ng/ml after 4-month follow-up. So timely intervention measures are very necessary for patients with ovarian cyst with abnormally elevated Tg level.

## Data Availability Statement

The original contributions presented in the study are included in the article/supplementary material. Further inquiries can be directed to the corresponding author.

## Ethics Statement

The studies involving human participants were reviewed and approved by the ethics committee of the First Affiliated Hospital of Anhui Medical University. The patients/participants provided their written informed consent to participate in this study.

## Author Contributions

TW, XZ, and HX performed image acquisition and post-processing and completed the manuscript. All authors contributed to the article and approved the submitted version.

## Funding

This work was supported by the National Nature Science Foundation of China (no. 81401448 and no. 81971643).

## Conflict of Interest

The authors declare that the research was conducted in the absence of any commercial or financial relationships that could be construed as a potential conflict of interest.
